# A Toolkit for Advancing Age Inclusivity in Higher Education

**DOI:** 10.1093/geroni/igab046.860

**Published:** 2021-12-17

**Authors:** Joann Montepare, Kimberly Farah

## Abstract

The pioneering Age-Friendly University (AFU) initiative, endorsed by GSA’s Academy for Gerontology in Higher Education (AGHE), calls for institutions of higher education to respond to shifting demographics and the needs of our aging populations through more age-friendly programs, practices, and partnerships. Over 70 institutions have joined the AFU global network and adopted the 10 AFU guiding principles. In support of the initiative, a GSA-AGHE-AFU workgroup was organized to develop strategies to help GSA members and their campuses explore how they can be more age-inclusive and create pathways to joining the AFU network. One outcome of the workgroup’s efforts was the creation “Tools for Advancing Age Inclusivity in Higher Education”, designed with support from AARP. In this symposium, workgroup members describe this suite of tools which can be used by faculty, students, administrators, and other campus leaders. Montepare will introduce the symposium with an overview of the AFU network and the workgroup’s goals. Morrow-Howell and Schumacher will discuss tools for “Making the Case” with examples from efforts on their campuses. Porter and Bergman will describe tools for “Getting Started” and how campuses can begin to mobilize age-friendly efforts. Andreoletti and June will share tools for “Gaining Momentum” with tips for creating age-friendly campus connections and collaborations. Silverstein and Gugliucci will describe tools for “Assessing and Tracking Success” that can be used at any stage of the process for exploring a campus’s age-friendliness. Information about joining the AFU network will be provided.

